# Analysis of mode of delivery according to race and ethnicity in Brazil: Application of the Robson Classification

**DOI:** 10.1002/ijgo.70720

**Published:** 2025-12-04

**Authors:** Fernanda Rafaella Correa dos Santos, Fernanda Garanhani Surita, Maria Laura Costa, José Paulo Siqueira Guida

**Affiliations:** ^1^ Department of Gynecology and Obstetrics University of Campinas Campinas Brazil

**Keywords:** birth, cesarean delivery, delivery, racial disparities

## Abstract

**Objective:**

Cesarean section rates are one indicator of obstetric care quality. While low rates might reflect limited access to timely obstetric interventions, excessively high rates are not associated with better maternal or perinatal outcomes. Racial and ethnic disparities in maternal outcomes raise concerns about the equitable distribution of obstetric interventions in Brazil. The aim of this study was to analyze cesarean section rates across racial and ethnic groups in Brazil from 2012 to 2022, using the Robson Ten‐Group Classification System.

**Methods:**

This cross‐sectional study used public data from the Live Birth Monitoring Panel, maintained by the Brazilian Ministry of Health. All hospital births from 2012 to 2022 were included. Deliveries were stratified by Robson group and self‐reported race/color: White, Black, Mixed‐race (Parda), Indigenous, and Asian. For each group, we calculated the proportion of total births, C‐section rates, and contribution to overall cesarean numbers. Prevalence ratios were calculated using White women as the reference group.

**Results:**

A total of 28 998 245 births were analyzed; 56.44% were cesarean deliveries. Cesarean rates were highest among White women (66.66%) and lowest among Indigenous women (21.23%). Robson Group 5 was the most prevalent and contributed the most to cesarean rates in all racial groups. Cesarean rates were consistently lower among Black, Mixed‐race, Indigenous, and Asian women, across nearly all Robson groups.

**Conclusion:**

Marked racial disparities exist in cesarean rates in Brazil. These differences might reflect unequal access to obstetric interventions and raise concerns about equity in maternal health care.

## 1 INTRODUCTION

Cesarean section rates are one of the various possible indicators of the quality of obstetric care. While low cesarean rates might reflect barriers to accessing appropriate obstetric care, with potential adverse effects on maternal and perinatal outcomes, excessively high rates have not been associated with improved outcomes.^1^ To date, no ideal range for cesarean rates has been validly established across different contexts of obstetric transition and economic development that both ensures perinatal health and avoids exposing women to unnecessary surgical procedures.^2^

When all childbearing women are considered as a single group, the overall cesarean rate provides limited insight. For this reason, the World Health Organization (WHO) endorsed the use of the Ten‐Group Classification System (TGCS), also known as the Robson Classification. This system is based on the principles of clinical relevance, ease of application, and being both totally inclusive and mutually exclusive, ensuring that all persons delivering can be appropriately categorized.^3^, ^4^, ^5^

The TGCS stratifies women based on five obstetric characteristics: parity, onset of labor, gestational age, fetal presentation, and number of fetuses. This allows the estimation of the size of each group, the cesarean rate within each group, and each group's contribution to the overall number of cesarean deliveries. Box 1 presents the 10 groups in this system.^4^

BOX 1 **Description of the Ten Groups Classification System (also known as Robson's Classification)**

**Table:** 

Group	Description
1	Nulliparous, single fetus, cephalic presentation, ≥37 weeks, spontaneous labor
2	Nulliparous, single fetus, cephalic presentation, ≥37 weeks, induced labor or cesarean before labor
3	Multiparous without a previous cesarean, single fetus, cephalic presentation, ≥37 weeks, spontaneous labor
4	Multiparous without a previous cesarean, single fetus, cephalic presentation, ≥37 weeks, induced labor or cesarean before labor
5	Multiparous with at least one previous cesarean, single fetus, cephalic presentation, ≥37 weeks
6	All nulliparous, single fetus, breech presentation
7	All multiparous, single fetus, breech presentation (including those with previous cesarean)
8	All multiple pregnancies (including those with previous cesarean)
9	All single fetus, transverse or oblique lie (including those with previous cesarean)
10	All single fetus, cephalic presentation, <37 weeks (including those with previous cesarean)

This classification system provides a standardized framework for analyzing obstetric populations and cesarean delivery rates. This table was adapted from Robson et al.

While low cesarean rates in such populations might indicate barriers to appropriate obstetric interventions, with potential adverse effects on maternal and perinatal outcomes, excessively high rates in other groups have not been associated with improved results.^6^ The WHO advocates that, rather than aiming for a specific cesarean rate, the primary goal should be to ensure that no woman is denied the intervention when it is medically necessary, as lack of access to cesarean sections might even result in preventable deaths.^2^

Brazil is undergoing its obstetric transition; that is, a period in which a significant proportion of maternal deaths can be prevented, and the maternal mortality ratio can be consistently reduced through the implementation of interventions aimed at decreasing deaths from hemorrhage, hypertension, and infections.^7^, ^8^ In Brazil, maternal mortality is disproportionately higher among Black, mixed‐race (“Parda” is the Brazilian Portuguese name), and Indigenous women compared to White women. This persistent disparity likely reflects structural inequities in the quality and timeliness of obstetric care.^9^, ^10^, ^11^

The mechanisms by which racial and socioeconomic inequities influence obstetric decision‐making and the use of cesarean delivery remain poorly understood.^12^ Understanding differences for cesarean section in different racial and ethnic groups might contribute to the ongoing debate on optimal cesarean use, helping to identify points of intervention to improve obstetric care, especially among marginalized populations. This study aimed to investigate cesarean section rates across different racial and ethnic groups among deliveries in Brazil.

## 2 METHODS

### 2.1 Study design

This is a cross‐sectional study that includes all births in Brazil between 2012 and 2022. Data were obtained from the Live Births Monitoring Panel, an Integrated Health Surveillance platform maintained by the Brazilian Ministry of Health.

We included in our study all deliveries for which information on the mother's race/ethnicity, as well as classification according to Robson groups, was available. Deliveries lacking information on ethnic characteristics or not classified within the Robson groups were excluded from this analysis.

### 2.2 Data collection

The data used in this study are publicly accessible via the internet, through the Live Births Monitoring Panel, available at https://svs.aids.gov.br/daent/centrais‐de‐conteudos/paineis‐de‐monitoramento/natalidade/nascidos‐vivos/. The selected filters included “Reference Year,” with successive selection of each year from 2012 to 2022. The second filter was “Race/Color,” with successive selection of the categories defined by the Brazilian Institute of Geography and Statistics (IBGE): White, Black, Pardo, Indigenous, and Asian. The third filter applied was “Robson Group,” with data extracted for each of the 10 groups. Finally, the filter “Type of Delivery” was used, distinguishing between vaginal delivery and cesarean section.

The sampling was based on convenience, including all deliveries available following the abovementioned procedures for the data collection time interval (2012 until 2022).

The extracted data were compiled into a Microsoft Excel database, categorized by number of births and number of cesarean deliveries for each TGCS group, for each year, and according to race/color. These data were subsequently reviewed by a second researcher who was not involved in the initial data extraction process.

### 2.3 Primary data source

The primary data source for the Live Births Monitoring Panel is the “Live Birth Certificate” (Declaração de Nascido Vivo). This document is mandatory for every birth occurring in Brazilian territory and is required for issuing a birth certificate and any official document attesting to Brazilian citizenship. This document is essential for accessing any social and health rights in Brazil, such as vaccination and access to primary care. Therefore, virtually all births in Brazil are recorded through this document.

The certificate is completed in three copies, one of which is automatically forwarded to the local municipality. The data are then transferred to the state government, which verifies and consolidates the information before transmitting it to the Ministry of Health, which is responsible for aggregating, anonymizing, and publicly distributing the data.

The use of this database for scientific research has been common in studies conducted in Brazil, given its near‐complete coverage of deliveries across the country. This allows for accurate and reliable data, since, as described above, the information undergoes three stages of verification before being made publicly available.^13^, ^14^

The classification of race in Brazil is particularly complex due to the widespread phenomenon of miscegenation. The IBGE categorizes the population as White, Pardo, Black, Indigenous, and Asian. This classification is based on self‐identification. The term “Pardo” corresponds to “mixed‐brown” in English and is not considered pejorative or discriminatory in the Brazilian context. We chose to use the current use in Brazilian Portuguese in this article (“Pardo”) because there are no equivalent terms in English.

### 2.4 Statistical analysis

The extracted data were analyzed according to the World Health Organization (WHO) recommendations for evaluating TGCS groups. The total number of births and cesarean deliveries was calculated for each group to determine the size of each group, the cesarean section rate within each group, and the absolute and relative contributions of each group to overall cesarean deliveries. This same analysis was then repeated for each racial/ethnic group, using the same methodology.

In the final step, cesarean section rates in each Robson group were compared using prevalence ratios between each racial/ethnic group and the White women group, which was chosen as the reference category. In the Brazilian social context, White women tend to experience fewer socioeconomic vulnerabilities and, consequently, have greater access to quality health care. A 95% confidence interval was calculated for each of the obtained prevalence ratios. The statistical analysis was performed using the software EpiInfo 7.2.6.0.

### 2.5 Ethical considerations

This study used only public data that are freely and openly accessible. No individual‐level data were assessed; only aggregated data were analyzed using the filters described above. Therefore, as there was no access to or manipulation of individual data, Brazilian ethical regulations waive the need for individual consent or prior review by a Research Ethics Committee for this type of study.

## 3 RESULTS

During the study period, 28 998 245 births were included, of which 16 366 761 were cesarean deliveries, yielding an overall cesarean rate of 56.44%. Group 5 was the most prevalent (6 504 379 women, 22.43%), followed by Groups 3 (5 410 698 women 18.66%), 1 (5 160 852 women, 17.80%), and 2 (4 561 391 women, 15.73%). Group 5 accounted for 19.0% of all cesarean deliveries, followed by Group 2 (11.04%) and Group 1 (8.14%). Group 10 accounted for 10.19% of the study population, with a cesarean rate of 51.05%, representing 5.20% of the total cesareans during the study period. These results are presented in Table 1.

**TABLE 1:** Distribution of deliveries in Brazil from 2012 until 2022 according to the Ten‐Groups Classification System.

Group	Number of CS in group	Number of women in group	Group size (%)	Group CS rate (%)	Absolute group contribution to overall CS rate	Relative contribution of group to overall CS rate
1	2 361 396	5 160 852	17.80%	45.76%	8.14%	14.43%
2	3 202 747	4 561 391	15.73%	70.21%	11.04%	19.57%
3	1 026 628	5 410 698	18.66%	18.97%	3.54%	6.27%
4	1 235 479	2 723 082	9.39%	45.37%	4.26%	7.55%
5	5 568 672	6 504 379	22.43%	85.61%	19.20%	34.02%
6	387 022	427 615	1.47%	90.51%	1.33%	2.36%
7	484 414	559 022	1.93%	86.65%	1.67%	2.96%
8	525 192	627 150	2.16%	83.74%	1.81%	3.21%
9	66 573	68 716	0.24%	96.88%	0.23%	0.41%
10	1 508 638	2 955 340	10.19%	51.05%	5.20%	9.22%
Total	16 366 761	28 998 245	100.00%	56.44%		100.00%

Abbreviation: CS, cesarean section.

Among the included women, 28 885 743 (99.61%) had data available on self‐declared race/color at the time of delivery. Of these, 16 315 417 (56.48%) identified as Pardo, 10 545 552 (36.51%) as White, 1 756 369 (6.08%) as Black, 256 353 (0.89%) as Indigenous, and 12 052 (0.04%) as Asian.

Table 2 presents the group sizes for each Robson group by race and color, showing differences among the considered groups. We observed that group 5 was the largest among White women (2 707 377 women, 25.67%) and Asian women (27 641 women, 22.19%). Among Black (366 242 women, 20.42%), Pardo (3 643 502 women, 22.33%), and Indigenous (112 751 women, 43.98%) women, group 3 was the largest.

**TABLE 2:** Absolute number and frequency of women (group size) by Robson group and race/color in Brazil, 2012–2022.

Robson group	White	Black	Brown	Indigenous	Asian	Total
1	1 619 087 (15.35%)	279 103 (15.89%)	3 198 290 (19.60%)	43 627 (17.02%)	20 745 (16.66%)	5 160 852 (17.80%)
2	2 254 632 (21.38%)	263 446 (15.00%)	2 008 172 (12.31%)	11 451 (4.47%)	23 690 (19.02%)	4 561 391 (15.73%)
3	1 268 332 (12.03%)	366 242 (20.85%)	3 643 502 (22.33%)	112 751 (43.98%)	19 871 (15.95%)	5 410 698 (18.66%)
4	1 020 517 (9.68%)	199 497 (11.36%)	1 478 056 (9.06%)	12 333 (4.81%)	12 679 (10.18%)	2 723 082 (9.39%)
5	2 707 377 (25.67%)	363 605 (20.70%)	3 379 451 (20.71%)	26 305 (10.26%)	27 641 (22.19%)	6 504 379 (22.43%)
6	195 399 (1.85%)	20 474 (1.17%)	207 906 (1.27%)	1647 (0.64%)	2189 (1.76%)	427 615 (1.47%)
7	204 343 (1.94%)	32 933 (1.88%)	315 979 (1.94%)	3428 (1.34%)	2339 (1.88%)	559 022 (1.93%)
8	262 340 (2.49%)	41 875 (2.38%)	316 394 (1.94%)	3493 (1.36%)	3048 (2.45%)	627 150 (2.16%)
9	25 839 (0.25%)	3968 (0.23%)	37 867 (0.23%)	742 (0.29%)	300 (0.24%)	68 716 (0.24%)
10	987 686 (9.37%)	185 226 (10.55%)	1 729 800 (10.60%)	40 576 (15.83%)	12 052 (9.68%)	2 955 340 (10.19%)
Total	10 545 552 (100.00%)	1 756 369 (100.00%)	16 315 417 (100.00%)	256 353 (100.00%)	124 554 (100.00%)	28 998 245 (100.00%)

Table 2 presents the absolute frequency of cesarean section and the respective cesarean section rates in Robson's group, according to race and color. The results showed that cesarean section rates were higher among White women in all 10 Robson's groups, as well as in the overall cesarean section rate. In contrast, Indigenous women had lower cesarean section rates for all Robson's groups. These lower rates were observed even in groups 6 to 9, in which the expected cesarean section rates were higher than the observed.

**FIGURE 1:**
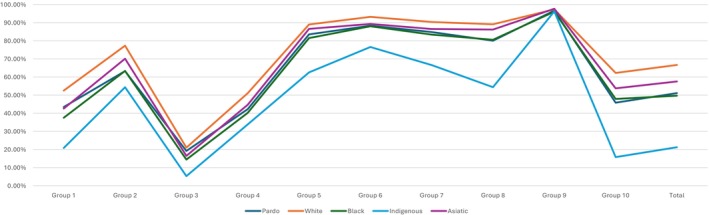
Cesarean section rates in the Robson's Group according to skin color in Brazil.

Figure 1 summarizes cesarean section rates in Robson's groups by skin color; more detailed data are presented in the Supporting Information Tables S1–S5, which include results for each group.

Among women identified as Parda, the overall cesarean rate was 51.11%. The most prevalent group was Group 3 (22.33%), followed by Groups 5 (20.71%), 1 (19.60%), and 2 (12.31%). Group 5 accounted for 17.30% of all cesareans, followed by Group 1 (8.51%). Group 10 accounted for 10.60% of the sample, contributing 4.86% of the total cesareans.

White women showed an overall cesarean rate of 66.66%. Among these women, Group 5 was the most prevalent (25.67%) and accounted for the most significant proportion of cesareans (22.85%). It was followed by Groups 2 (21.38%), 1 (15.35%), and 3 (12.03%). Group 2 was the second largest contributor to cesarean deliveries, accounting for 16.52%.

Among Black women, the cesarean rate was 49.69%. The largest groups were Group 3 (20.85%), Group 5 (20.70%), Group 1 (15.89%), and Group 2 (15.00%). Group 5 accounted for 16.87% of cesareans, followed by Group 2 with 9.51%. Group 10 accounted for 10.55% of the population, with a cesarean rate of 47.89%.

Indigenous women had the lowest cesarean rate, at 21.23%. Group 3 was the largest (43.98%), followed by Groups 1 (17.02%), 10 (15.83%), and 5 (10.26%). Group 5 contributed the most to the absolute number of cesareans (6.42%), followed by Group 1 (3.54%).

Among women of Asian descent, the cesarean rate was 57.54%. Group 5 accounted for 22.19% of women, followed by Groups 2 (10.02%), 1 (16.66%), and 3 (15.95%). Group 5 accounted for 19.21% of all cesarean deliveries, followed by Group 2 at 13.33%.

**FIGURE 2:**
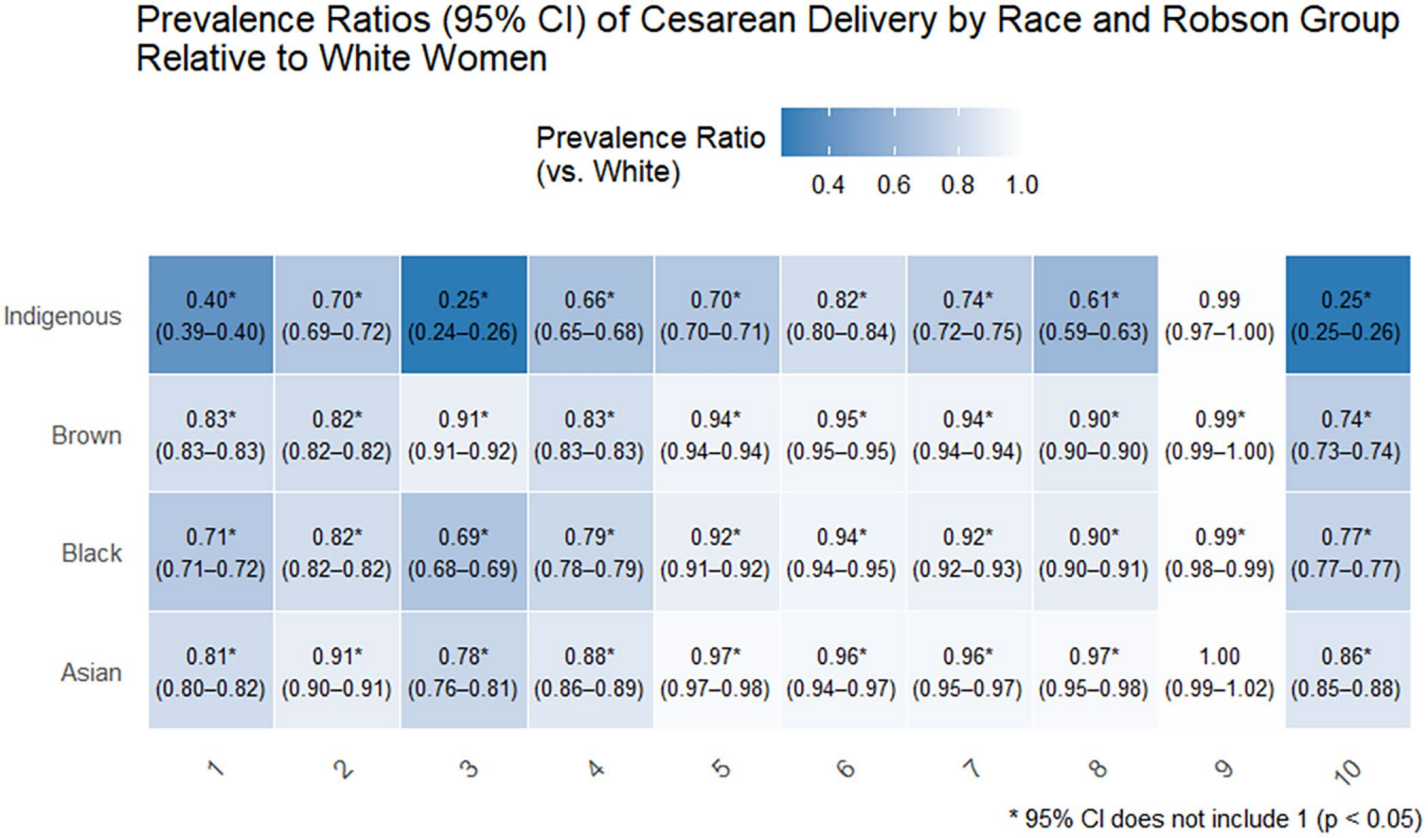
Prevalence ratios (95% CI) of Cesarean Delivery by Race and Robson Group Relative to White Women.

Figure 2 presents a heat map showing the prevalence ratios of cesarean section by race and Robson's group in comparison to White women. Groups represented in dark blue had higher cesarean section rates, while those in lighter blue were closer to the prevalence presented in the group of White women. The figure shows that Indigenous and Black women had significantly lower cesarean section rates than those observed among White women. Analysis by Robson group revealed that Indigenous women had low cesarean section rates, especially if they were in groups 1, 3, and 10; a similar effect was observed among Black women (Table 3).

**TABLE 3:** Absolute number and frequency of cesarean section (cesarean rates) by Robson group and race/color in Brazil, 2012–2022.

Robson group	White	Black	Brown	Indigenous	Asian	Total
1	849 766 (52.48%)	104 676 (37.50%)	1 389 047 (43.43%)	9078 (20.81%)	8829 (42.56%)	2 361 396 (45.76%)
2	1 742 601 (77.29%)	166 958 (63.37%)	1 270 354 (63.26%)	6225 (54.36%)	16 609 (70.11%)	3 202 747 (70.21%)
3	266 355 (21.00%)	52 812 (14.42%)	698 250 (19.16%)	5940 (5.27%)	3271 (16.46%)	1 026 628 (18.97%)
4	520 949 (51.05%)	80 224 (40.21%)	624 471 (42.25%)	4171 (33.82%)	5664 (44.67%)	1 235 479 (45.37%)
5	2 409 358 (88.99%)	296 296 (81.49%)	2 822 642 (83.52%)	16 450 (62.54%)	23 926 (86.56%)	5 568 672 (85.61%)
6	182 152 (93.22%)	18 019 (88.01%)	183 635 (88.33%)	1261 (76.56%)	1955 (89.31%)	387 022 (90.51%)
7	184 681 (90.38%)	27 468 (83.41%)	267 960 (84.80%)	2282 (66.57%)	2023 (86.49%)	484 414 (86.65%)
8	233 733 (89.10%)	33 725 (80.54%)	253 208 (80.03%)	1899 (54.37%)	2627 (86.19%)	525 192 (83.74%)
9	25 152 (97.34%)	3812 (96.07%)	36 602 (96.66%)	714 (96.23%)	293 (97.67%)	66 573 (96.88%)
10	614 702 (62.24%)	88 698 (47.89%)	792 362 (45.81%)	6400 (15.77%)	6476 (53.73%)	1 508 638 (51.05%)
Total	7 029 449 (66.66%)	872 688 (49.69%)	8 338 531 (51.11%)	54 420 (21.23%)	71 673 (57.54%)	16 366 761 (56.44%)

## 4 DISCUSSION

This study evaluated a Brazilian national database containing 28 998 245 births from 2012 to 2022. Of these, the vast majority (99.61%) had available information on race and color, allowing the estimation of cesarean rates across different ethnic and racial groups using the Ten‐Group Classification System. Further, it showed that cesarean rates were consistently lower among Indigenous, Black, and Parda women when compared to White women, across all 10 groups.

The overall cesarean rate was 56.44%, and among White women, this figure reached 66.66%; among Indigenous women, the cesarean rate was 21.23%. Lower cesarean rates were observed among Black, Parda, Indigenous, and Asian women. When considering the increased maternal mortality ratios among Black, Parda, and Indigenous women, this result might suggest a disparity in the provision of cesarean sections to non‐White women, with potential impact on their maternal outcomes.

The prevalence of maternal death among Black and Indigenous Brazilian women is higher than that observed among White women, a finding that remains consistent even after considering other variables such as age, region of residence, and cause of death.^9^, ^11^ Our results do not allow us to determine whether the lack of access to cesarean delivery was a decisive factor for the occurrence of deaths; however, the limited access to cesarean sections might partly explain the higher maternal mortality rates observed among Black and Indigenous women.

Robson Group 5 (multiparous women with previous cesarean, singleton, cephalic fetus, ≥37 weeks) was the largest group across all racial groups, also being the main contributor to cesarean sections performed. This finding reinforces the importance of preventing the first cesarean because undergoing it substantially increases the probability of subsequent cesareans.^15^

The evaluation of Brazilian data showed that among Black women, there were fewer deliveries in the lithotomy position and fewer episiotomies performed, which could suggest that Black women were exposed to better obstetric care. However, the finding that they also received less analgesia and less support for early breastfeeding suggests that Black women experienced childbirth with less assistance, especially considering that practices such as episiotomy are routine in many Brazilian hospitals. These findings reinforce the perception that the lower cesarean rate among Black women is not evidence of quality obstetric care but rather a lack of adequate access to health services.^16^

Other obstetric outcomes might also show racial disparities in their occurrence; one example is the frequency of anal sphincter injuries, which are more prevalent in different types of delivery (vaginal or operative) among Asian women. A higher frequency of such injuries was also observed among Black women in their first delivery when exposed to the use of forceps.^17^

Maternal‐request cesarean sections are those for which there is no medical recommendation for their performance. A model that adjusted for different confounding variables, such as clinical conditions, age, income, and education, showed that in the United States, Black, Hispanic, Asian, and Indigenous women had less access to maternal‐request cesareans, suggesting a racial disparity in access to the procedure, which is more easily accessible to White women.^18^

In the Brazilian context, the recommendation is that maternal‐request cesareans be performed from 39 weeks onward, and there are regions in the country where this is considered a legal right. It is possible that greater access to maternal‐request cesarean sections among White women also occurs in the Brazilian population. Our data, however, do not allow for the identification of the reason for the cesarean indication.

Post‐cesarean care involves the rapid identification of signs of postoperative complications, as well as the adequate administration of analgesic medications, according to the individual's pain threshold. Among Asian and Black women, lower administration of analgesics was observed after cesarean section, even after the implementation of “Enhanced Recovery After Surgery” (ERAS) protocols.^19^

Recognizing that pain during labor and recovery is an individual phenomenon—and must, therefore, be treated according to each person's needs—places health professionals in a position where they can make a difference in the care setting. Thus, it is essential to acknowledge potential biases in healthcare, which result in reduced access to care for vulnerable populations.^20^

Postpartum care also showed disparities between Black and Hispanic women compared to White women, especially during periods of healthcare system disruption, such as the COVID‐19 pandemic.^21^ Therefore, this fact must be taken into account, especially in contexts where cesarean rates are high, ensuring that women have access to postpartum monitoring as well as effective contraception to determine their reproductive future.

The main limitations of this study include the use of a database that does not allow for individual‐level data analysis or verification of individual record integrity. Thus, it was not possible to perform multivariate analyses or adjust the results considering the multiple variables included. Further, although race and color were theoretically based on self‐identification, it is possible that in some cases this procedure was not followed, and race/color classification was made based on the perception of the person responsible for completing the birth declaration. In contrast, the large number of cases included likely reduced the impact of such limitations.

## 5 CONCLUSION

This population‐based study revealed substantial racial disparities in cesarean section rates in Brazil from 2012 to 2022. The use of the Robson Classification showed that White women had significantly higher cesarean rates in nearly all obstetric groups compared to Indigenous, Black, and Pardo women—suggesting barriers to access rather than optimal care in the latter populations.

## AUTHOR CONTRIBUTIONS

FRCdS performed data collection, reviewed data consistency, and wrote the first version of the draft. FGS and MLC reviewed the results and the first version of the draft. JPSG performed statistical analysis, reviewed the results, and wrote the first version of the draft. All authors approved the final version of the article.

## CONFLICT OF INTEREST STATEMENT

The authors declare no conflicts of interest.

## Supporting information


**Table S1.** Distribution of deliveries among Pardo women in Brazil from 2012 until 2022 according to the Ten‐Groups Classification System.
**Table S2.** Distribution of deliveries among White women in Brazil from 2012 until 2022 according to the Ten‐Groups Classification System.
**Table S3.** Distribution of deliveries among Black women in Brazil from 2012 until 2022 according to the Ten‐Groups Classification System.
**Table S4.** Distribution of deliveries among Indigenous women in Brazil from 2012 until 2022 according to the Ten‐Groups Classification System.
**Table S5.** Distribution of deliveries among Asian women in Brazil from 2012 until 2022 according to the Ten‐Groups Classification System.

## Data Availability

Data sharing is not applicable to this article as no new data were created or analyzed in this study.
