# Supplementary material for: Analysis of mode of delivery according to race and ethnicity in Brazil: Application of the Robson Classification
Source: Int J Gynaecol Obstet. 2025 Dec 4;173(2):1087–93. doi: 10.1002/ijgo.70720 (PMC13094671; doi:10.1002/ijgo.70720)
Supplement: Supplementary file 1 — Table S1. Distribution of deliveries among Pardo women in Brazil from 2012 until 2022 according to the Ten‐Groups Classification System. Table S2. Distribution of deliveries among White women in Brazil from 2012 until 2022 according to the Ten‐Groups Classification System. Table S3. Distribution of deliveries among Black women in Brazil from 2012 until 2022 according to the Ten‐Groups Classification System. Table S4. Distribution of deliveries among Indigenous women in Brazil from 2012 until 2022 according to the Ten‐Groups Classification System. Table S5. Distribution of deliveries among Asian women in Brazil from 2012 until 2022 according to the Ten‐Groups Classification System. [file IJGO-173-1087-s001.docx]

| **Supplemental Table 1 – Distribution of deliveries among Pardo women in Brazil from 2012 until 2022 according to the Ten-Groups Classification System.** | | | | | | |
| --- | --- | --- | --- | --- | --- | --- |
| Grupo | Number of CS in group | Number of women in group | Group size (%) | Group CS rate (%) | Absolute group contribution to overall CS rate | Relative contribution of group to overall CS rate |
| 1 | 1389047 | 3198290 | 19,60% | 43,43% | 8,51% | 16,66% |
| 2 | 1270354 | 2008172 | 12,31% | 63,26% | 7,79% | 15,23% |
| 3 | 698250 | 3643502 | 22,33% | 19,16% | 4,28% | 8,37% |
| 4 | 624471 | 1478056 | 9,06% | 42,25% | 3,83% | 7,49% |
| 5 | 2822642 | 3379451 | 20,71% | 83,52% | 17,30% | 33,85% |
| 6 | 183635 | 207906 | 1,27% | 88,33% | 1,13% | 2,20% |
| 7 | 267960 | 315979 | 1,94% | 84,80% | 1,64% | 3,21% |
| 8 | 253208 | 316394 | 1,94% | 80,03% | 1,55% | 3,04% |
| 9 | 36602 | 37867 | 0,23% | 96,66% | 0,22% | 0,44% |
| 10 | 792362 | 1729800 | 10,60% | 45,81% | 4,86% | 9,50% |
| Total | 8338531 | 16315417 | 100,00% | 51,11% |  | 100,00% |

| **Supplemental Table 2 – Distribution of deliveries among White women in Brazil from 2012 until 2022 according to the Ten-Groups Classification System.** | | | | | | |
| --- | --- | --- | --- | --- | --- | --- |
| Grupo | Number of CS in group | Number of women in group | Group size (%) | Group CS rate (%) | Absolute group contribution to overall CS rate | Relative contribution of group to overall CS rate |
| 1 | 849766 | 1619087 | 15,35% | 52,48% | 8,06% | 12,09% |
| 2 | 1742601 | 2254632 | 21,38% | 77,29% | 16,52% | 24,79% |
| 3 | 266355 | 1268332 | 12,03% | 21,00% | 2,53% | 3,79% |
| 4 | 520949 | 1020517 | 9,68% | 51,05% | 4,94% | 7,41% |
| 5 | 2409358 | 2707377 | 25,67% | 88,99% | 22,85% | 34,28% |
| 6 | 182152 | 195399 | 1,85% | 93,22% | 1,73% | 2,59% |
| 7 | 184681 | 204343 | 1,94% | 90,38% | 1,75% | 2,63% |
| 8 | 233733 | 262340 | 2,49% | 89,10% | 2,22% | 3,33% |
| 9 | 25152 | 25839 | 0,25% | 97,34% | 0,24% | 0,36% |
| 10 | 614702 | 987686 | 9,37% | 62,24% | 5,83% | 8,74% |
| Total | 7029449 | 10545552 | 100,00% | 66,66% |  | 100,00% |

| **Supplemental Table 3 – Distribution of deliveries among Black women in Brazil from 2012 until 2022 according to the Ten-Groups Classification System.** | | | | | | |
| --- | --- | --- | --- | --- | --- | --- |
| Grupo | Number of CS in group | Number of women in group | Group size (%) | Group CS rate (%) | Absolute group contribution to overall CS rate | Relative contribution of group to overall CS rate |
| 1 | 104676 | 279103 | 15,89% | 37,50% | 5,96% | 11,99% |
| 2 | 166958 | 263446 | 15,00% | 63,37% | 9,51% | 19,13% |
| 3 | 52812 | 366242 | 20,85% | 14,42% | 3,01% | 6,05% |
| 4 | 80224 | 199497 | 11,36% | 40,21% | 4,57% | 9,19% |
| 5 | 296296 | 363605 | 20,70% | 81,49% | 16,87% | 33,95% |
| 6 | 18019 | 20474 | 1,17% | 88,01% | 1,03% | 2,06% |
| 7 | 27468 | 32933 | 1,88% | 83,41% | 1,56% | 3,15% |
| 8 | 33725 | 41875 | 2,38% | 80,54% | 1,92% | 3,86% |
| 9 | 3812 | 3968 | 0,23% | 96,07% | 0,22% | 0,44% |
| 10 | 88698 | 185226 | 10,55% | 47,89% | 5,05% | 10,16% |
| Total | 872688 | 1756369 | 100,00% | 49,69% |  | 100,00% |

| **Supplemental Table 4 – Distribution of deliveries among Indigenous women in Brazil from 2012 until 2022 according to the Ten-Groups Classification System.** | | | | | | |
| --- | --- | --- | --- | --- | --- | --- |
| Grupo | Number of CS in group | Number of women in group | Group size (%) | Group CS rate (%) | Absolute group contribution to overall CS rate | Relative contribution of group to overall CS rate |
| 1 | 9078 | 43627 | 17,02% | 20,81% | 3,54% | 16,68% |
| 2 | 6225 | 11451 | 4,47% | 54,36% | 2,43% | 11,44% |
| 3 | 5940 | 112751 | 43,98% | 5,27% | 2,32% | 10,92% |
| 4 | 4171 | 12333 | 4,81% | 33,82% | 1,63% | 7,66% |
| 5 | 16450 | 26305 | 10,26% | 62,54% | 6,42% | 30,23% |
| 6 | 1261 | 1647 | 0,64% | 76,56% | 0,49% | 2,32% |
| 7 | 2282 | 3428 | 1,34% | 66,57% | 0,89% | 4,19% |
| 8 | 1899 | 3493 | 1,36% | 54,37% | 0,74% | 3,49% |
| 9 | 714 | 742 | 0,29% | 96,23% | 0,28% | 1,31% |
| 10 | 6400 | 40576 | 15,83% | 15,77% | 2,50% | 11,76% |
| Total | 54420 | 256353 | 100,00% | 21,23% |  | 100,00% |

| **Supplemental Table 5 – Distribution of deliveries among Asian women in Brazil from 2012 until 2022 according to the Ten-Groups Classification System.** | | | | | | |
| --- | --- | --- | --- | --- | --- | --- |
| Grupo | Number of CS in group | Number of women in group | Group size (%) | Group CS rate (%) | Absolute group contribution to overall CS rate | Relative contribution of group to overall CS rate |
| 1 | 8829 | 20745 | 16,66% | 42,56% | 7,09% | 12,32% |
| 2 | 16609 | 23690 | 19,02% | 70,11% | 13,33% | 23,17% |
| 3 | 3271 | 19871 | 15,95% | 16,46% | 2,63% | 4,56% |
| 4 | 5664 | 12679 | 10,18% | 44,67% | 4,55% | 7,90% |
| 5 | 23926 | 27641 | 22,19% | 86,56% | 19,21% | 33,38% |
| 6 | 1955 | 2189 | 1,76% | 89,31% | 1,57% | 2,73% |
| 7 | 2023 | 2339 | 1,88% | 86,49% | 1,62% | 2,82% |
| 8 | 2627 | 3048 | 2,45% | 86,19% | 2,11% | 3,67% |
| 9 | 293 | 300 | 0,24% | 97,67% | 0,24% | 0,41% |
| 10 | 6476 | 12052 | 9,68% | 53,73% | 5,20% | 9,04% |
| Total | 71673 | 124554 | 100,00% | 57,54% |  | 100,00% |
